# Cerebrovascular function and cognition in childhood: a systematic review of transcranial doppler studies

**DOI:** 10.1186/1471-2377-14-43

**Published:** 2014-03-06

**Authors:** Mireille J Bakker, Jessica Hofmann, Owen F Churches, Nicholas A Badcock, Mark Kohler, Hannah AD Keage

**Affiliations:** 1 Cognitive Neuroscience Laboratory, School of Psychology, Social Work and Social Policy, University of South Australia, GPO BOX 2471, 5001 Adelaide, SA, Australia; 2 Department of Cognitive Neuroscience, Donders Institute for Brain, Cognition and Behavior, Radboudumc, Nijmegen, The Netherlands; 3 Brain and Cognition Laboratory, School of Psychology, Flinders University, Adelaide, Australia; 4 ARC Centre of Excellence in Cognition and its Disorders, Department of Cognitive Science, Macquarie University, Sydney, Australia

**Keywords:** Cognition, Infants, Children, Adolescents, Transcranial doppler, Cerebrovascular

## Abstract

**Background:**

The contribution of cerebrovascular function to cognitive performance is gaining increased attention. Transcranial doppler (TCD) is portable, reliable, inexpensive and extremely well tolerated by young and clinical samples. It enables measurement of blood flow velocity in major cerebral arteries at rest and during cognitive tasks.

**Methods:**

We systematically reviewed evidence for associations between cognitive performance and cerebrovascular function in children (0-18 years), as measured using TCD. A total of 2778 articles were retrieved from PsychInfo, Pubmed, and EMBASE searches and 25 relevant articles were identified.

**Results:**

Most studies investigated clinical groups, where decreased blood flow velocities in infants were associated with poor neurological functioning, and increased blood flow velocities in children with Sickle cell disease were typically associated with cognitive impairment and lower intelligence. Studies were also identified assessing autistic behaviour, mental retardation and sleep disordered breathing. In healthy children, the majority of studies reported cognitive processing produced lateralised changes in blood flow velocities however these physiological responses did not appear to correlate with behavioural cognitive performance.

**Conclusion:**

Poor cognitive performance appears to be associated with decreased blood flow velocities in premature infants, and increased velocities in Sickle cell disease children using TCD methods. However knowledge in healthy samples is relatively limited. The technique is well tolerated by children, is portable and inexpensive. It therefore stands to make a valuable contribution to knowledge regarding the underlying functional biology of cognitive performance in childhood.

## Background

The neural underpinnings of cognition have received a great deal of attention in the developmental literature. Neuronal activity is coupled with blood supply in the brain [1], and given this, the role of cerebrovascular function in relation to cognitive function in childhood is an important consideration [2-4]. Cerebrovascular function in childhood has been investigated using a variety of techniques such as functional magnetic resonance imaging (fMRI), positron emission tomography (PET) and near infrared spectroscopy (NIRS). However, these techniques are expensive and there are feasibility issues which are particularly problematic in child samples, including requirements of sitting or lying still for a prolonged duration [5].

Recently, Transcranial Doppler (TCD) ultrasonography has been increasingly employed as a non-invasive, inexpensive, safe and portable technique for measuring cerebrovascular function. It permits continuous and bilateral recording of cerebral blood flow velocity through the major intracranial vessels (middle, anterior, posterior and basilar arteries), is relatively resistant to movement artefact, has good test-retest reliability [4], and is a suitable technique for child samples [3,4,6,7]. Further, given that the skull is relatively thin in childhood (most notably around the temporal window), blood vessels are easily insonated and failure to detect a signal is rare, unlike in aged populations where skull thickness can impede recording in around 30 percent of cases [8]. Measurements can be taken at rest and during cognitive tasks. TCD data collected during cognitive operations is referred to as functional TCD (fTCD), and is a calculation of the average increase in blood flow velocity (over multiple trials) relative to a specific mental operation [9].

Given that the contribution of cerebrovascular function to cognition is important and that the technique is well suited to child populations, we aimed to systematically review the literature to assess how TCD measures relate to cognitive performance in childhood. In doing so, we wanted to highlight measures which appear to correlate with cognitive performance (and those which do not), and point to key areas of future research.

## Methods

### Review criteria

Primary and secondary screens of the literature were performed. A primary screen, including PubMed, Embase, and PsycINFO searches, was conducted for peer-reviewed papers published between January 1970 and 31 May 2013. The following search terms were employed in the primary screen: (ultrasonography doppler transcranial OR TCD OR transcranial doppler*) AND (baby OR newborn* OR infant* OR preschool* OR teenager* OR child* OR adoles*). A total of 2778 articles were retrieved.

Titles and abstracts were read by at least two of the authors (MJB, JH or HADK). Articles were retained if they collected TCD data at rest or during a cognitive task, from any cerebral artery accessible via TCD (e.g. anterior, middle, posterior or basilar) and where the mean age of participants was less than or equal to 18 years of age. Bibliographies from the identified articles were also manually searched for relevant publications. Articles were excluded if they were not written in English, were a case-report, or did not look at the statistical association between a TCD measure and a measure of cognitive performance. Preferred Reporting Items for Systematic Reviews and Meta-Analyses (PRISMA) guidelines were followed [10].

### Data extraction

If the article was included, the following data was extracted: participant characteristics (i.e. number, age range or mean if unavailable, male/female ratio, clinical diagnosis if applicable), TCD protocol and vessel, cognitive measure, and relevant findings (linking TCD and cognition results).

## Results

**Figure 1:**
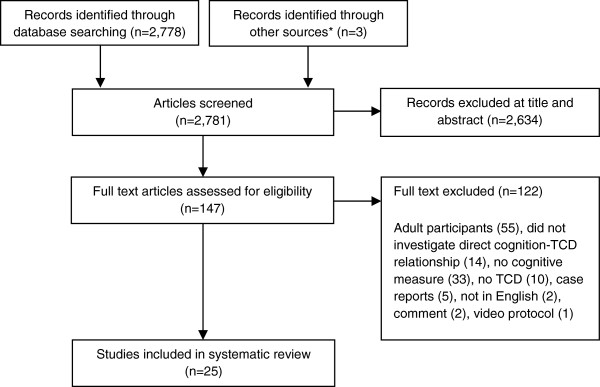
**Preferred Reporting Items for Systematic Reviews and Meta-Analyses (PRISMA) flow diagram of study identification.** * Through references of manual search.

**Figure 2:**
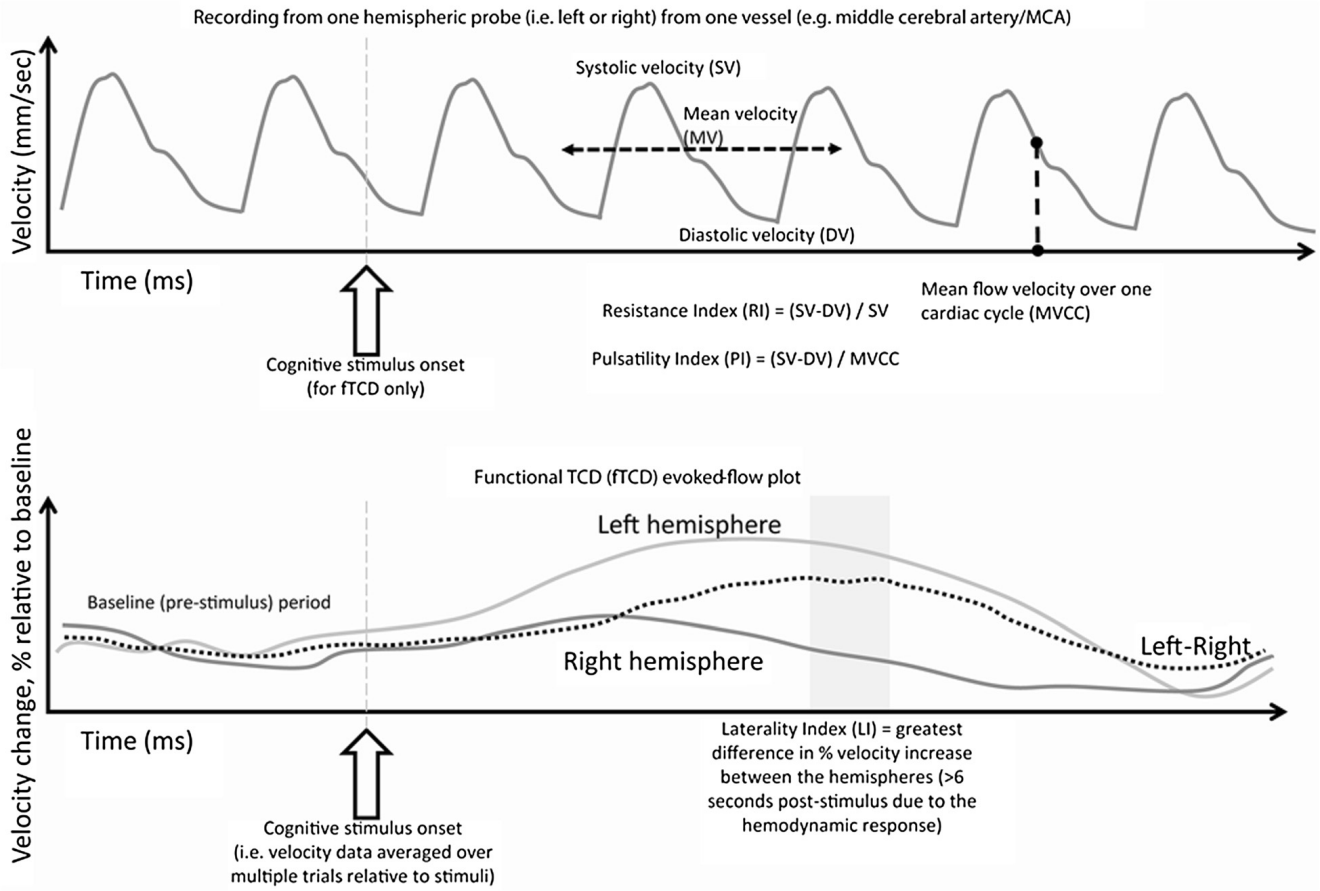
**Top: recording from one hemispheric probe in one vessel including calculation of TCD metrics: systolic peak flow velocity, diastolic velocity, mean flow velocity, resistance index and pulsatility index.** Bottom: functional TCD (fTCD) evoked-flow plot including calculation of laterality index.

Twenty-five peer-reviewed research articles were eligible for this review, representing data from 1693 participants. Publication dates ranged from 1 January 1991 to 31 May 2013. A flow diagram of study identification is shown in Figure 1. Methodological characteristics of each study are summarized in Table 1 and include: study sample (including sex, age and diagnoses), TCD protocol and vessel(s) investigated, cognitive measure, and key finding (i.e., those that relate to an association between a cognitive measure and a TCD measure). The calculation of common resting TCD measures employed in identified studies are summarised in Figure 2 including: systolic peak flow velocity (SV), end diastolic velocity (DV), mean flow velocity (MV), the pulsatility index (PI), and resistance index (RI). The fTCD evoked-flow response, and resultant measures, is also displayed in Figure 2.

**Table 1:** Summary of the articles using resting and functional/cognitive TCD measures to investigate the association between blood flow velocity and cognition in children

**Article**	**Participants v controls (if applicable)**	**Total N (subgroups)/mean age (sd)/% males**	**TCD protocol and vessel(s)**	**Cognitive measure**	**Relevant findings**
***RESTING TCD STUDIES***					
***In infants***					
Rennie et al. [13]	Low birth weight infants with developmental delay (DD) v normal development infants	n = 74 (DD n = 9, control n = 42)/mean gestational age weeks DD 27 (SD = 1), control 29 (SD = 2)/DD 67% male, control 50% male	Resting TCD; MV recorded over first 3 days of life	BSID (mental development) taken at 18 months of age.	DD infants less likely to show the usual steady increase in SV. A larger percentage (44%) of DD infants displayed a rise, then a fall, in CBFV compared to infants in the control group (13%).
			Unilateral ACA		
Ojala et al. [12]	Ventilated preterm infants with RDS (V-RDS) and non-ventilated preterm infants with no signs of RDS (N-RDS)	n = 49 (V-RDS n = 35, N-RDS n = 14)/V-RDS mean gestational age 29 weeks (SD = 1), N-RDS mean gestational age 31 weeks (SD = 1)/V-RDS 57% males, N-RDS 57% male	Resting TCD; SV, DV, MV and RI taken at 6, 12, and 24 hours post-birth	10-min Apgar score.	In N-RDS group, lower RI was associated with poorer neurologic results at 12 months. No association in V-RDS group.
				GMDS (physical and mental development) at 12 months.	
			Unilateral ACA		
Arditi et al. [11]	Premature infants	n = 51/mean gestational age weeks 30 (SD = 3)/53% male	Resting TCD; SV, DV and MV	NBAS (visual/auditory habituation) at 37 weeks gestational age.	Greater right SV related to lower neonatal performance on the NBAS. Greater left SV related to high MDI scores at 24 months. Left > right SV related to better neonatal orientation and high MDI scores at 24 months.
			Bilateral MCA		
				BSID-II (mental development) at 6, 12, and 24 months.	
Scherjon et al. [14]	Premature infants	n = 123/mean gestational age 31 weeks/sex not reported	Resting TCD; MV and MV ratio (defined as MV during the first 12 hours after birth, divided by mean velocity in the period 12-hours post-birth)	Prechtl score (neurological functioning) at 40 weeks corrected gestational age.	Prechtl scores were related to MV ratio – high Prechtl score related to low MV ratio (reflecting MV not increasing over first days of life). No relationships between Touwen scores and TCD measures.
				Touwen score (neurological functioning) at ages 6 and 12 months.	
			MCA		
Alatas et al. [15]	Infants from HRP mothers v infants from normal pregnancy mothers	n = 237 (HRP n = 75, control n = 162)/mean gestational age at delivery HRP 38 weeks, control 39 weeks/sex not reported	In-utero from 24 weeks gestation until delivery	1 and 5-minute Apgar scores (general and neurological functioning).	Positive relationship between MCA PI and 5-min Apgar scores in infants from HRP; no relationship in control group.
			MCA		
***In Sickle Cell Disease***					
Hogan et al. [19]	Infants with SCD v healthy infants	n = 28 (SCD n = 14, control n = 12)/between 1-13 months/SCD 79% male, control 42% male	Resting TCD; SV and MV recorded at ages 3, 9 and 12 months	BINS (neurological function) taken at 3, 9 and 12 months.	Infants with SCD had increased SV and MV, which were associated with a higher risk of neurodevelopmental delay at 9 months of age (not 3 or 12 months).
			BA, bilateral MCA and ICA		
Armstrong et al. [30]	Infants with SCD	n = 208/mean age 13 months (SD = 3)/43% male	Resting TCD; SV	BSID-II (mental, motor and behavioural development), VABS (developmental status based on parent report).	Lower VABS scores (communications, daily living skills and socialisation domains) were associated with greater SV. No association between SV and BSID-II.
			Vessel not specified		
Schatz et al. [25]	Children with SCD	n = 50/mean age 26 months (SD = 13)/56% male	Resting TCD; MV	DDST-II (infant and childhood development).	Children with developmental delays had significantly higher MCA MVs, compared typically developing children (all with SCD).
			Bilateral ICA and MCA		
		Note: TCD in an n = 30 subsample		VABS (developmental status based on parent report)	
Aygun et al. [16]	Children with SCD	n = 88/mean age 4 years (SD < 1)/51% male	Resting TCD; MV	BPS-II (motor, language and cognitive development).	No association was found between TCD measures and cognitive performance.
			BA, bilateral MCA, ACA, PCA and ICA		
Sanchez et al. [24]	Children with SCD	n = 39/mean age 6 years (SD = 1)/41% male	Resting TCD; SV	TOLD-P:3 (language ability).	Higher SVs were associated with poorer syntactical ability.
			Bilateral MCA	WJ-III (academic achievement).	
Strouse et al. [26]	Children with SCD	n = 24/mean age 9 years (SD = 2)/sex not reported	Resting TCD; SV	WASI (IQ).	No significant correlation between SV and IQ.
			Bilateral ACA		
Onofri et al. [31]	Children with SCD	n = 35/mean age 9 years (SD = 3)/49% male	Resting TCD; SV	WISC-II (IQ) for children aged 6-16 years.	There were no differences between mentally impaired and non-impaired groups in those terms of percentage with abnormal SV.
			Abnormal SV defined as >170 cm/s		
			Vessel unspecified	WPPSI-III (IQ) for children aged 4-6 years.	
Kral et al. [22]	Children with SCD	n = 60/mean age 121 months (SD = 31)/43% male	Resting TCD; SV	WASI (IQ).	Children with abnormal TCD had lower verbal IQs than children with conditional TCD. Children with conditional TCD performed worse than children with normal TCD on measures of executive function. The conditional TCD group was slowest to complete the TMT.
			Abnormal TCD defined as SV > 200 cm/sec; conditional TCD defined as SV = 170-200 cm/sec; normal TCD defined as SV <170 cm/sec	WJ-R (academic achievement).	
				CPT-II (visual sustained attention).	
				CMS (working memory).	
			Bilateral MCA and ICA	TMT (visual attention).	
				BRIEF (executive functioning).	
Kral and Brown [20]	Children with SCD	n = 62/mean age 121 months (SD = 31)/43% male	Resting TCD; MV	Academic attainment; grade retention and special education placement.	Children with abnormal TCD received significantly more special education services.
			Abnormal TCD defined as SV > 200 cm/sec		
			Bilateral MCA and ICA		
Kral et al. [21]	Children with SCD	n = 27/mean age 129 months (SD = 36)/44% male	Resting TCD; MV	WASI (IQ).	When controlled for age and hematocrit, children with abnormal TCD had better verbal memory in comparison to children with normal TCD.
			Abnormal TCD defined as SV > 200 cm/sec; normal TCD defined as SV < 170 cm/sec	WJ-R (academic achievement).	
				CPT-II (sustained attention),	
			Bilateral MCA, ACA and ICA	CMS (working memory).	
				TMT (visual attention).	
				Academic attainment; grade retention and special education placement.	
				DTVMI (visuo-motor integration).	
Hijmans et al. [18]	Children with severe SCD	n = 37/mean age 12 years (SD = 3)/53% male	Resting TCD; MV (within 14 months preceding neurocognitive testing)	WISC-III/WAIS-III (IQ),	No associations between TCD and cognitive measures. However, sustained attention better in children with right > left resting MV.
				Stop task (response inhibition and sustained attention), Tower of London (planning),	
			Bilateral MCA, ACA and ICA		
				N-back task; (working memory).	
Bernaudin et al. [17]	Children with SCD v siblings without SCD	n = 249 (SCD n = 173, control n = 76)/mean age SCD 10 years (SD = 3), not reported for controls/SCD 51% male, controls not reported	Resting TCD; SV, DV and MV	WISC-II/WPPSI-R (IQ).	SCD sample: children with abnormal TCD had lower IQ scores (picture arrangement and performance IQ).
			Abnormal TCD defined as SV > 200 cm/sec		
			BA, bilateral MCA, ACA, PCA, and ICA		
					Effect did not hold after excluding those with stroke.
		TCD only collected on a n = 143 subsample			
Ruffieux et al. [23]	Children with SCD	n = 32/between 6 and 24 years of age/48% male	Resting TCD; SV and DV	CVLT (memory). Executive function/attention tasks including	Children with A-TCD had worse memory performance, as compared to children with N-TCD measurements.
			Normal (N-TCD) defined as max. velocity <170 cm/sec; abnormal (A-TCD) defined as max. velocity >200 cm/sec or peak systolic ≥ 250 cm/sec		
				Colour trails, digit span, coding, verbal semantic fluency test, bell cancellation task, letter-number sequencing and CPT.	
			OA, MCA, ACA, BA, ICA		
***In sleep disordered breathing***					
Hill et al. [17]	Children with mild SDB v healthy control children	n = 31 (SDB n = 21, control n = 17)/mean age SBD 6 years (SD = 1), control 5 years (SD = 1)/SDB 43% male, control 53% male	Resting TCD; SV, MV	WPPSI-III (IQ).	CBFV significantly increased in the SDB group, as compared to controls. SDB children achieved lower scores on processing speed and visual attention, but no direct association with CBFV.
			Bilateral MCA	NEPSY (neuropsychological development).	
				BRIEF (executive functioning).	
***FTCD STUDIES***					
***In clinical groups***					
Bruneau et al. [27]	Children with autistic behaviour (AB), mentally retarded children without autistic symptoms (MR) and healthy control children	n = 34 (AB n = 12, MR n = 10, control n = 12)/mean age AB 7 years (SD = <1), MR 6 years (SD < 1), control 7 years (SD < 1)/AB 67%, MR 70%, control 33% male	fTCD using auditory stimuli (passive task); SV, DV, MV and RI	Auditory tone stimuli	Auditory stimulation increased velocities and decreased RI on the left side for the control children. Same pattern for MR children, but less asymmetrical. AB children displayed a symmetric and opposite pattern, with velocities decreasing and RI increasing on both sides.
			Bilateral MCA		
***In typically developing children***					
Stroobant et al. [32]	Typically developing children	n = 26/mean age 82 months/46% male	fTCD using visual stimuli requiring expressive response, and auditory stimuli requiring no response; MV and calculated LI (left-right MV in response to stimuli)	*Taaltest voor kinderen/“*language tests for children” (language development)	The expressive language task elicited a stronger left LI than the passive language task. Good test-retest reliability for TCD. No relationship between LI and Dutch language test.
			Bilateral MCA		
Lohmann et al. [4]	Typically developing children	n = 10/2-10 years of age/40% male	fTCD with a picture description task; MV and calculating LI (left-right MV in response to stimuli)	Illinois Test of Psycholinguistic	Left lateralised increased in blood flow velocity (i.e. positive lateralisation index), however this did not relate to language ability.
				Abilities	
			Bilateral MCA		
Groen et al. [2]	Typically developing children	n = 14/mean age 7 years (SD < 1)/57% male	fTCD using visual stimuli requiring a physical response; MV and calculated LI (left-right MV in response to stimuli)	Rabbits paradigm (visuospatial memory)	A negative LI was found, indicating right lateralisation. Task performance unrelated to LI.
			Bilateral MCA		
Groen et al. [3]	Typically developing children	n = 60/6-16 years of age/43% male	fTCD using visual stimuli requiring a verbal and physical response; MV and calculated LI (left-right MV in response to stimuli)	LIPS-R (performance IQ).	Most children (58%) were left lateralised for language, and right lateralised for visuospatial memory. Children with left lateralisation language had better vocabulary and non-word reading performance (independent of laterality of visuospatial memory).
				BPVS-2 (receptive vocabulary).	
				TOWRE (reading). NEPSY (phonological short-term memory).	
			Bilateral MCA		
Haag et al. [28]	Typically developing right handed children	n = 45 (children n = 23, adolescents n = 22)/mean age children 8 years (SD = 2), adolescents 15 years (SD = 2)/children 48% males, adolescents 36% males	fTCD using visual stimuli requiring a verbal response; MV and calculated LI (left-right MV in response to stimuli)	Visual stimuli.	The world generation task (adolescents only) produced left lateralised response, but this was not the case for the picture description task, regardless age group.
				Children completed a non-lexical picture description task. Adolescents additionally completed a lexical word generation task	
			Bilateral MCA		

Vessels: ACA Anterior cerebral artery, BA Basilar artery, ICA Internal cerebral artery, MCA Middle cerebral artery, OA Ophthalmic artery, PCA Posterior cerebral artery, RCP Regional cerebral perfusion.

Health Conditions: HRP High risk pregnancy, RDS Respiratory distress syndrome, SCD Sickle cell disease, SDB Sleep disordered breathing, HLHS Hypoplastic left heart syndrome.

Neurocognitive Measures: NBAS Neonatal Behaviour Assessment Scales. BINS Bayleys Infant Neurodevelopmental Screener, BSID Bayley Scales of Infant Development, BSID-II Bayley Scales of Infant Development-II, BSITD-III Bayleys Scales of Infant and Toddler Development-III, BRIEF Behaviour Rating Inventory of Executive Functioning, BPS-II Brigance Preschool Screen-II, BPVS-II British Picture Vocabulary Scale-II, CVLT California Verbal Learning Test for Children, CMS Children's Memory Scale, CPT/CPT-II Conners's Continuous Performance Test-II, DDST-II Denver Developmental Screening Test, GMDS Griffith’s Mental Development Scale, LIPS-R Leiter International Performance Scale-Revised, NEPSY Neuropsychological Test Battery for Children, TOLD-P:3 Test of Language Development-Primary Third Edition, TOWRE Test of Word Reading Efficiency, TMT Trail Making Test, WASI Wechsler Abbreviated Scale of Intelligence, WAIS-III Wechsler Adult Intelligence Scale-III, WISC-II/WISC-III Wechsler Intelligence Scale for Children, WPPSI-III/WPPSI-R Wechsler Preschool and Primary Scale of Intelligence, WJ-R/WJR-III Woodcock-Johnson Psycho-Educational Battery, VABS Vineland Adaptive Behaviour Scales.

Nineteen (of the 25) papers assessed TCD measures during rest, and six employed fTCD (i.e. changes in velocity relative to a cognitive operation). The vast majority investigated clinical samples (20 of 25): including preterm infants [11-14], infants from high risk pregnancy [15], Sickle cell disease [16-26], autism spectrum disorders [27], and sleep disordered breathing [7]. Most of these studies did not employ a healthy comparison group, instead looking at associations within the clinical group. The remaining studies assessed TCD-cognition relationships (5 of 25 articles) in typically developing children [2-4,28,29].

For resting TCD, a number of measures were used, including mean velocity, systolic velocity, diastolic velocity, pulsatility index and resistance index, typically measured bilaterally from the MCA, ICA or ACA. Less commonly the PCA and BA were assessed. Six articles employing fTCD were included, all measuring bilaterally from the MCA [2-4,27-29]. A broad range of behavioural and cognitive domains were assessed such as the intelligence quotient (IQ; including the Wechsler Intelligence Scale for Children, Wechsler Preschool and Primary Scale of Intelligence and the Woodcock-Johnson Psycho-Educational Battery), neonatal behaviour (e.g. Neonatal Behaviour Assessment Scales), language (e.g. Test of Language Development-Primary Third Edition and British Picture Vocabulary Scale-II, Test of Word Reading Efficiency), memory (e.g. Children's Memory Scale and the California Verbal Learning Test for Children) and executive function (e.g. Conners's Continuous Performance Test and Trail Making Test). All studies are summarised in Table 1. Major findings are discussed below, relative to clinical (Sickle cell disease and others) and non-clinical child samples.

### Children with Sickle cell disease

There were thirteen studies investigating children with Sickle cell disease, with most reporting an association between high blood flow velocities and impaired cognition, from infanthood to adolescence. Hogan et al. [19] reported that in infants (assessed at nine months) with Sickle cell disease, high blood flow velocities (both systolic velocity and mean velocity in the BA, MCA and ICA) were associated with moderate to high risk of neurodevelopmental delay. Armstrong et al. [30] reported that lower scores in the communications, daily living and socialisation domains of the Vineland Adaptive Behaviour Scales were associated with higher systolic velocities, even for velocities within the normal range in infants with Sickle cell disease. Similarly, Schatz et al. [25] reported that preschool-aged children with Sickle cell disease and delayed development had significantly higher MCA mean velocities, compared to healthy controls. Sanchez et al. [24] reported a negative association between MCA systolic velocities and syntactical ability in children with Sickle cell disease. Furthermore, Ruffieux et al. [23] reported an association between abnormally high velocities (maximum velocity >200 cm/sec or peak systolic >250 cm/sec) and poor memory performance in children with Sickle cell disease.

Kral et al. [22] found that children and adolescents with Sickle cell disease and abnormal systolic velocities (defined as maximum velocity >200 cm/sec in the MCA and ICA) had lower verbal IQ scores than those with conditional/mid-range systolic velocity (defined as maximum velocity 170-200 cm/sec). Children displaying these mid-range systolic velocities performed worse than children with normal systolic velocities (defined as maximum velocity <170 cm/sec) on measures of sustained attention/concentration and executive function. The conditional systolic velocities group were however the slowest to complete the Trail Making Test. Employing the same sample, Kral and Brown [20] reported that these laboratory measures of cognitive function did not translate to problems with everyday measures of cognition (i.e. academic attainment and grade retention). However, children with abnormally high TCD measures received significantly more special education services [20].

With respect to sample size, the largest study (n = 143) included in this review was performed by Bernaudin and colleagues [17] and involved children with Sickle cell disease and non-affected siblings. Children with Sickle cell disease and abnormal TCD (defined as maximum velocity >200 cm/sec in the BA, MCA, ACA, PCA and ICA) had lower cognitive performance than children with Sickle cell disease and normal TCD for Picture Arrangement and Performance IQ (on the WISC-II/WPPSI-R). However, no significant difference was observed after the exclusion of participants with a history of stroke (n = 5).

In the only study to report a positive association between blood flow velocity and cognition in a Sickle cell disease sample, Kral et al. [21] reported that after controlling for age and hematocrit, children with Sickle cell disease and high and mid-range systolic velocities had better verbal memory (Children’s Memory Scale Stories) compared with children with Sickle cell disease and normal TCD velocities. There were also reports of a lack of association between blood flow velocities and cognition in Sickle cell disease child samples [16,18,26,31]. Onofri et al. [31] found no relationship between IQ (performance and verbal) and systolic velocities in a sample of children with Sickle cell disease. A study by Aygun et al. [16] observed no association between scores on an academic screening test and mean velocities (from multiple vessels). Strouse and colleagues [26], reported no correlation between ACA systolic velocities and IQ in children with Sickle cell disease. Similarly, Hijmans et al. [18] reported no association between mean velocity (MCA, ACA, and ICA) and cognitive measures (including IQ, response inhibition, sustained attention, planning and visuospatial working memory) in children with severe Sickle cell disease. However, children with right greater than left asymmetries in resting mean velocities had better sustained attention [18].

### Children with other clinical conditions

Alatas and colleagues [15] reported that in babies from high-risk pregnancies there was a positive relationship between in-utero MCA pulsatility index and five-minute Apgar scores after delivery, where low pulsatility index was associated with poor physical and neurological health. There was no association in babies from mothers with normal pregnancies.

Three studies were conducted in premature infants [11,12,14]. Scherjon et al. [14] reported that in preterm infants without intracranial haemorrhage, there was a relationship between MCA mean velocity ratio (defined as mean velocity in the first 12 hours after birth, divided by mean velocity in the period from 12 hours until 168 hours after birth) and Prechtl score at 40 weeks corrected gestational age; where if MCA mean velocity did not increase after birth, the child was more likely to have a suspected neurological abnormality. They reported no relationships between mean velocity ratio and neurological functioning at six and 12 months [14]. Arditi and colleagues [11] reported that in a group of infants born prematurely with TCD data collected at corrected 37 weeks gestational age: (1) higher right MCA systolic velocities were related to poorer neonatal perceptual performance (on habituation and orientation); (2) higher left MCA systolic velocities were associated with a better Mental Development Index score at 24 months; and (3), those with higher left MCA systolic velocities (as compared to right MCA systolic velocities) had better neonatal orientation and habituation scores as neonates and MDI scores at 24 months. In another study of preterm infants, Ojala and colleagues [12] compared those being ventilated for respiratory distress syndrome (V-RDS) with preterm infants with no signs and not ventilated (N-RDS) using the ACA resistance index. They reported that during the early postnatal transition (the first day of life measured at six, 12 and 24 hours), a lower resistance index was associated with a higher risk of adverse neurologic outcome in the group of N-RDS preterm infants at 12 months of age; this was not the case for the V-RDS infants.

Rennie et al. [13] found that developmentally delayed infants (defined as a score of <70 on the Bayley Scale of Infant Development at age 18 months) did not show the usual steady increase in ACA mean velocity over the first three days of life, as compared to typically developing infants. A rise then a fall in mean velocity occurred in four of nine of the developmentally delayed group (44%), whereas this pattern occurred in a smaller percentage (four of 31 participants, 13%) of the group who were subsequently normal.

Bruneau et al. [27] compared children with autistic behaviour, mentally retarded children without autistic symptoms and typically developing controls. In the healthy control group they found that auditory stimulation resulted in increased left MCA mean velocity and decreased left MCA resistance index. This pattern was also observed in the mentally retarded without autistic symptoms group, although the degree of lateralisation was reduced. However, in the autistic group, children displayed a symmetrical decrease of MCA mean velocities and an increase in the resistance index.

Hill and colleagues [7] reported that MCA systolic velocity was significantly higher, processing speed slower, and visual attention poorer, in a group of children with mild sleep disordered breathing as compared to controls. However, these cognitive and TCD measures did not significantly correlate.

### Typically developing samples

Five studies assessed healthy children in order to investigate relationships between fTCD and cognition using visual stimulation [2,3], passive and expressive language tasks [4,29], and (non-) lexical word generation paradigm [28]. A recent study by Groen et al. [3] reported left-lateralized activation for language production and right-lateralized activation for visuospatial memory in the majority of children (58%). In this study, boys showed a trend for stronger right-hemisphere lateralization for visuospatial memory than girls, but there was no gender effect on language laterality. Children with left-lateralized language production achieved higher standard scores on vocabulary and non-word reading, regardless of the laterality of visuospatial memory. Having language and visuospatial functions in the same hemisphere was not associated with poor cognitive performance [3]. In a previous study, Groen et al. [2] reported a right hemispheric specialization for a visuospatial task in children, however the degree of lateralisation was unrelated to cognitive performance.

Using a sample of right-handed children, Stroobant et al. [32] reported that both receptive and expressive language tasks elicited left hemispheric lateralization which was more pronounced in the expressive task. However, lateralization did not relate to language performance or hand dominance. Similarly, Lohmann et al. [4] reported that a language task induced a left-lateralised velocity increase in children between two and nine years, however this lateralisation was unrelated to language performance. Haag et al. [28] reported that in right-handed children and adolescents, the picture description paradigm failed to indicate left hemispheric dominance in a substantial proportion of the sample regardless of age. However, although Haag et al. [28] measured the effect of a cognitive process on blood flow velocity, they did not correlate changes with a behavioural cognitive measure.

## Discussion

We aimed to systematically review evidence for associations between cognitive performance and cerebrovascular function in children via TCD measures. Generally, high blood flow velocities were associated with poor cognitive performance in child Sickle cell disease samples, and decreased blood flow velocities were associated with poor cognitive outcomes in infants from high risk pregnancies or births. In healthy samples, blood flow-velocity increases were seen during cognitive tasks, with predicted lateralised responses seen for visual and verbal tasks, however these physiological responses did not appear to relate to standardised cognitive measures.

### Resting blood flow velocities and cognitive/neurological functioning

Resting cerebral blood flow velocities such as systolic, diastolic and mean were commonly assessed in relation to cognitive performance [7,11-14,16-26,30]. The majority of studies reported an association between blood flow velocity and cognition, with the extremes of systolic and mean blood flow velocities (i.e., very low or very high) being associated with poorer cognitive or neurological functioning. However, six studies reported no associations, including four Sickle cell disease studies [16,18,26,31], one study on preterm infants [12] and one on children with sleep disordered breathing [7]. The interpretation of these studies reporting no association is limited by the lack of information about potential confounders (e.g., medication doses and age), which are required to adequately examine the outcome of cognitive performance in children by means of TCD.

Much of the research reviewed here related to children with Sickle cell disease. Higher systolic and mean velocities were associated with lower IQ or poorer cognitive performance in seven samples of children with Sickle cell disease [17,19,22-25,30], although this effect did not hold in one sample when accounting for a history of stroke [17]. The reported cognitive deficits did not translate into academic achievement impairments, but those with higher systolic velocities did receive more special education services [20]. Conflicting other studies, Kral et al. [21] reported that after controlling for age and medication, children with Sickle cell disease and higher systolic velocities displayed better verbal memory performance than those with lower velocities. Four Sickle cell disease studies reported no association between cognitive performance and resting blood flow velocities [16,18,26,31]. There have been mixed findings from other brain imaging techniques in Sickle cell samples, for example poor cognitive function has been associated with decreased regional cerebral blood flow as measured using PET [33] and increased cerebral blood flow using fMRI [26].

Clinical studies assessing premature and low birth weight infants showed consistent associations between low blood flow velocities and poor cognitive outcomes. In a study tracking low birth weight infants, Rennie et al. [13] showed how over the first three days of life systolic velocity increased, and that infants with developmental delay at 18 months of age were less likely to show this characteristic increase. In a similar study of premature infants, Scherjon et al. [14] reported that a larger increase in mean velocity in the first days of life related to better neurological functioning at 40 weeks corrected gestational age. Likewise, another study found higher systolic velocities measured at birth were related to lower neonatal habituation performance and neurological functioning at two years [11].

There was some suggestion that asymmetries (i.e., differences between the hemispheres) in resting blood flow velocities were associated with cognitive performance. Greater left than right systolic velocity was related to better neonatal habitation performance and neurological functioning at two years [11]. Further, children with Sickle cell disease had better sustained attention when right mean velocities were greater than the left [18].

### Functional TCD measures and cognition/neurological functioning

The study by Bruneau et al. [27], also mentioned above, reported that blood flow velocities (mean, systolic and diastolic) increased during auditory stimulation in healthy and mentally retarded children, however velocities decreased in a group with autistic behaviours. They suggested that instead of downstream vasodilation in response to cognitive demands, those with autistic behaviours may vasoconstrict [27].

All other fTCD studies were in healthy samples [2-4,28,32]. In all studies a lateralised response during a cognitive operation was observed, with exception of one task (picture description, which was one of two tasks employed, the other being word generation) used by Haag et al. [28]. Stroobant et al. [32] noted that the expressive language task generated a greater lateralisation than the receptive language task. The lateralisation index (i.e., the difference in the degree of blood flow velocity increase between the hemispheres relative to a cognitive operation; see Figure 2) was related to cognitive performance in only one study [3], but not in four studies [2,4,28,32].

### Measures of vessel resistance and cognition/neurological functioning

Measures of vessel resistance such as pulsatility and resistance indexes have shown to relate to cognitive impairment in aged and demented samples [8]. These measures were only assessed in three childhood studies [12,15,27]. Low pulsatility index recorded in-utero was related to lower physical and neurological functioning five-minutes post-birth in infants from high risk pregnancies, but not in controls [15]. Similarly, low resistance index taken in the first day of life was related to neurological impairments at 12 months in a group of preterm infants who required ventilation after birth. However this effect was not found in preterm infants not requiring ventilation. It therefore appears that in preterm infants with compromised physical reserve, low cerebrovascular resistance puts them at risk for neurological impairment (at least temporarily).

Bruneau et al. [27] was the only study to assess changes in cerebrovascular resistance in relation to cognitive stimulation. They reported that the resistance index decreased on the left side during auditory stimulation in typically developing children, to a lesser extent in mentally retarded children, but increased in children with autistic behaviours [27].

### Limitations and future recommendations

One limitation of the TCD method is the assumption that artery diameter remains constant, therefore any change in velocity represents a change in flow. It has been reported however, that the diameter of the MCA does not significantly change during moderate alterations in blood pressure [34,35] and therefore any change in velocity reflects change in blood volume through the artery. Notably, and similar to other psychophysiological measurements, the evidence base for the reliability of TCD requires more work. In a recent paper, McDonnell et al. [36] reported that the test-retest reliability (three testing sessions over a few weeks) of the TCD-measured cerebrovascular response (CVR; change in blood flow velocity relative to inhalation of gas with 95% O_2_ and 5% CO_2_) was strong, particularly for measurements taken in sitting as opposed to supine. Sanchez et al. [24] reported elevated systolic velocities apparent in a group of five children with Sickle cell disease remained elevated over a six month period. These studies provide evidence for the reliability of TCD measures however further work is required, particularly in relation to fTCD measures.

Many studies did not detail the manufacturer of the TCD set-up employed. Specifications vary greatly between different manufacturers, for example sampling rates range from 1 Hz to 100 Hz. It is unknown if TCD measurements produced are equivalent between commercially available set-ups. Future papers must note the TCD manufacturer and associated recording parameters. Further, simple experimental design elements were often missing in identified studies, such as the time period used to collect resting TCD measures (e.g. 30 seconds, 1 minute or 2 minutes). A concerted effort to include these experimental design factors is required.

A further limitation of this review, and of all reviews of retrospectively published data, is that findings may be biased, with significant associations more likely to be published than non-significant associations [37]. Most studies investigated associations between cognition and TCD measures in Sickle cell disease samples, which limits extrapolation to other clinical as well as healthy populations. Future research therefore needs to focus on non-Sickle Cell disease samples. Further, age and sex effects have been under-investigated [9]. Of the articles reviewed here, one reported stronger right-hemisphere lateralization for visuospatial memory in boys as compared with girls [3]. In terms of assessing age-related change, TCD is particularly well placed to investigate changes in infancy, an age-range where other techniques (such as fMRI) are not feasible. Longitudinal analyses would provide opportunities for tracking the development of lateralisation of function. Recent analyses have shown that the relationship between the lateralisation index from fTCD and handedness are complex [38], deserving further assessment. Finally, neurovascular coupling has been investigated in adults simultaneously recording TCD and electroencephography [39,40]. This experimental design could be translated into child samples to investigate neurovascular coupling in healthy and clinical samples, and relationships with cognitive functioning.

Although all studies employed standardised cognitive and behavioural measures, they varied greatly, which made direct comparisons difficult. We were therefore unable to investigate how TCD measures differentially associate with cognitive domains. This would be a good avenue of future research.

## Conclusion

Poor cognitive performance appears to be associated with decreased blood flow velocities in premature infants, and increased velocities in Sickle cell disease children using TCD methods. TCD measures are sensitive to cerebrovascular demand during cognitive processing, with lateralised responses typical of the cognitive domain (i.e. visuospatial right and language left) assessed. There is an increased attention on vascular contributions to cognitive function and impairment, and interactions with neuronal function. TCD is a safe, portable, non-invasive method suitable for measurement of cerebrovascular function in child samples.

## Abbreviations

ACA: Anterior cerebral artery; BA: Basilar artery; MCA: Middle cerebral artery; PCA: Posterior cerebral artery; TCD: Transcranial doppler.

## Competing interests

All authors state no conflict of interest.

## Authors’ contributions

HADK conceptualised the study. MJB conducted the search and together with JH and HADK reviewed abstracts. MJB and HK extracted the data from identified publications. HADK and MJB drafted the manuscript. OFC, NAB, JH and MK revised the manuscript for important intellectual content. All authors read and approved the final manuscript.

## Pre-publication history

The pre-publication history for this paper can be accessed here:

http://www.biomedcentral.com/1471-2377/14/43/prepub
